# The Tadpole Pupil: Case Series With Review of the Literature and New Considerations

**DOI:** 10.3389/fneur.2019.00846

**Published:** 2019-08-19

**Authors:** Morgane Udry, Randy H. Kardon, Federico Sadun, Aki Kawasaki

**Affiliations:** ^1^FondationAsile des Aveugles, Department of Biology and Medicine, Hôpital Ophtalmique Jules Gonin, University of Lausanne, Lausanne, Switzerland; ^2^Department of Ophthalmology and Visual Sciences, Iowa City VA Center of Excellence for the Prevention and Treatment of Vision Loss, University of Iowa and Veterans Affairs Hospital, Iowa City, IA, United States; ^3^Ospedale Oftalmico Roma, ASL RM1, Rome, Italy

**Keywords:** pupil, tadpole pupil, pupillary distortion, iris dilator muscle, mydriasis, Horner syndrome

## Abstract

Tadpole pupil is a rare phenomenon in which segmental spasm of the iris dilator muscle results in a tadpole-shaped pupil. The pupillary distortion is usually unilateral, lasts several minutes, and can recur in clusters. Any segment of the iris can be affected; thus, for some patients, a different-shaped tadpole pupil is noticed from episode to episode. Tadpole pupil most commonly appears spontaneously in young women. Tadpole pupil is not associated with any systemic disorders, but an ipsilateral Horner syndrome is noted in 46% of patients. In this article, we have reviewed the existing literature of tadpole pupil, compiling all the published cases in a table and reporting four additional cases to re-examine the clinical profile of this disorder and to consider the different purported mechanisms as means to understand its possible etiology and treatment. The common denominator in the pathophysiology of tadpole pupil is a focal excessive contraction (segmental spasm) of the iris dilator muscle. Based on various proposed pathophysiologic mechanism of tadpole pupil, we can consider potential forms of treatment.

## Introduction

The term tadpole pupil refers to episodic, focal distortion of the pupillary shape. In 1983, Thompson et al. (1) presented the largest series of 26 patients and described the characteristic profile of tadpole pupil. It most commonly concerns young otherwise healthy women, arises spontaneously in one eye and disappears in 5 min or less. The tadpole pupil can be accompanied by a blurring of vision or an unusual sensation in the affected eye or on the ipsilateral side of the face. This segmental spasm of the iris dilator can involve any segment of the iris and is usually unilateral. In their series, two patients had bilateral simultaneous tadpole pupils during one episode and eight patients reported episodes of tadpole pupil that alternated sides. The episodes may recur several times a day for several days or weeks before subsiding spontaneously without leaving any sequelae. Between episodes, the pupil is round and reacts normally to light.

Since the seminal paper of Thompson et al. (1), individual reports of tadpole pupil have appeared in the literature. Various associations and mechanisms have been proposed. In this review, we have compiled all the published cases of tadpole pupil and added an additional four cases to re-examine its clinical profile and consider the different purported mechanisms as means to understand possible etiology and treatment. Written informed consent was obtained for each patient presented in the following paragraphs.

## Cases

### Case 1

**Figure 1:**
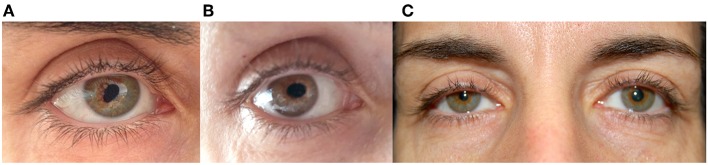
**(A,B)** Right eye of patient 1 during two episodes of tadpole pupil. Note the oval-shaped deformation of the pupil. **(C)** Between episodes, an anisocoria with a smaller right pupil and right upper lid ptosis was noted.

A 47-year-old healthy woman noted painless episodic distortion of her right pupil. The pupil became tear-shaped or oval-shaped then returned to a round shape within 1 min (Figures 1A,B). The peak of the tear shape was slightly different with each episode. These episodes occurred spontaneously several times daily in clusters of 2 to 3 days followed by pauses of weeks to months. The episodes have persisted for over 1 year. Recently, the patient noted right upper eyelid ptosis and a smaller right pupil (Figure 1C).

Ophthalmologic and neurologic examinations were normal except for anisocoria and right upper and lower lid ptosis. Instillation of one drop of 1% apraclonidine resulted in reversal of anisocoria and subtle retraction of the right upper lid, confirming a right oculosympathetic defect.

The patient's episodes of pupillary distortion were diagnosed as tadpole pupil. The anisocoria and lid ptosis were pharmacologically confirmed as a right Horner syndrome, which, though recently noticed by the patient, was apparent on old photographs about 1 year before the onset of tadpole pupil. Imaging studies of the head, neck, and chest were negative.

### Case 2

**Figure 2:**
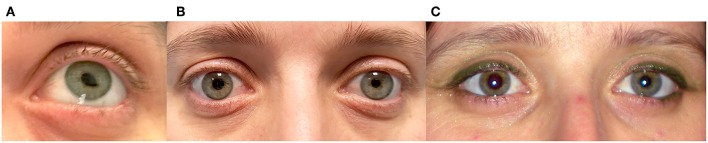
**(A)** Left eye of patient 2 during one episode of tadpole pupil. Note the tear-shaped deformation of the pupil. **(B)** Between episodes, examination showed an anisocoria with left pupillary miosis. **(C)** The anisocoria had been noticed by the patient for 15 years, as shown in this photograph taken 15 years previously.

A 41-year-old healthy woman noted painless episodic distortion of her left pupil (Figure 2A).The pupillary distortion occurred three to four times in 1 week and each episode lasted <1 min without other accompanying symptoms. No other episodes have occurred in the year since onset.

Past medical history revealed high myopia and myopic maculopathy of the right eye. She had undergone refractive surgery 11 years ago.

The patient had previously noticed a smaller pupil in her left eye. A review of old photographs dated this anisocoria to 15 years prior (Figure 2C). Ophthalmologic examination was normal except for an anisocoria with left pupillary miosis (Figure 2B). The palpebral fissures were symmetric. Instillation of topical 1% apraclonidine resulted in reversal of the anisocoria. A diagnosis of left tadpole pupil and a chronic left Horner syndrome was made.

### Case 3

**Figure 3:**
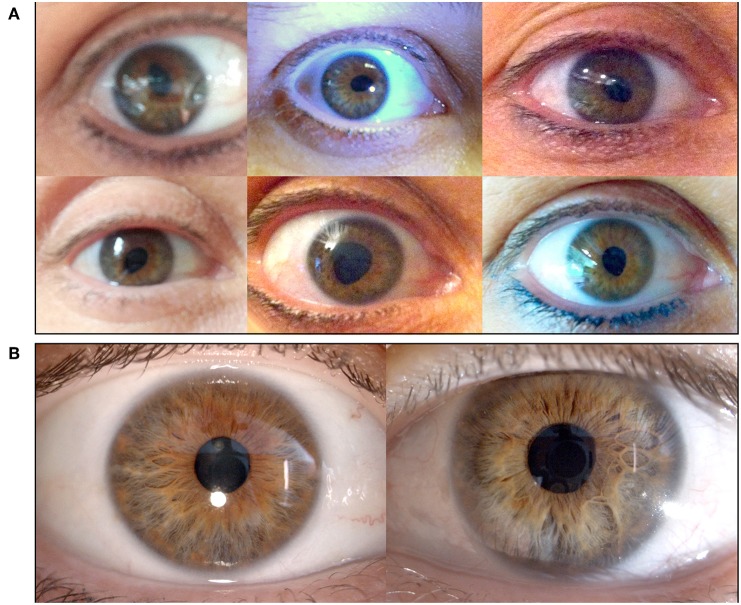
**(A)** Right eye of patient 3 during different episodes of tadpole pupil. This patient experienced painless episodic distortion of her right pupil accompanied by blurry vision. The pupillary distortion varied with each episode. **(B)** Between episodes, the patient demonstrated an anisocoria with a smaller right pupil; however, the larger left pupil was poorly reactive to light. Dilute pilocarpine testing (not shown) revealed cholinergic hypersensitivity of the left pupil indicating a left Adie pupil.

A 42-year-old healthy woman noted painless episodic distortion of her right pupil accompanied by blurry vision in her right eye. The episodes lasted 1 min or less. She also noted that the pupillary distortion frequently occurred in a different clock sector with each episode (Figure 3A). The episodes recurred weekly for 4 months, then became more occasional and resolved about 2 years after onset.

She also complained of dizziness upon standing in the past year and had had two syncopal episodes. There was no history of asymmetric or patchy sweating.

The neurologic and ophthalmologic examinations were normal except for anisocoria with right pupillary miosis (Figure 3B). The palpebral fissures were symmetric. No anhidrosis was noted. The left pupil demonstrated a poor constriction to light stimulation, and at the slit lamp, sectoral palsy of the iris sphincter muscle was observed. Instillation of dilute (0.125%) pilocarpine in both eyes caused left pupillary constriction consistent with cholinergic hypersensitivity of the left but not right pupil. Topical 1% apraclonidine had no effect on either pupil. A diagnosis of right tadpole pupil and left tonic (Adie) pupil was made.

### Case 4

A 32-year-old healthy man was presented with three to four episodes of spontaneous painless left pupillary distortion associated with mild visual blurring in the previous 2 months. Each episode lasted about 1 min. During one episode, he took a self-photograph and showed it to his neuroophthalmologist (FS), who confirmed a tadpole-shaped pupil. The episodes remitted several months and recurred.

His past medical history revealed only retinal laser treatment to both eyes for a retinal break 3 years previously.

Examination showed an anisocoria with left pupillary miosis and left upper lid ptosis (1 mm). The onset of anisocoria could not be confirmed from old photographs. Testing with topical apraclonidine did not show any dilation of the smaller left pupil, and the anisocoria remained unchanged. A diagnosis of left tadpole pupil was made. While a concomitant left Horner syndrome was suspected on clinical grounds, sympathetic denervation hypersensitivity was not demonstrated.

## Discussion

To search the literature about tadpole pupil, we used the Medline (PubMed) using the term “tadpole pupil,” “pupillary distortion,” and “iris dilator spasm” without restriction of publication date. We also reviewed the reference lists from retrieved articles to find articles not revealed by database search. Abstracts from meeting proceedings were not included.

Tadpole pupil is an episodic pupillary distortion resulting in a tadpole-shaped pupil. It is a rare condition, first described in 1912 by Erlenmeyer (2) and termed by Thompson et al. in 1983 (1). At present, only 43 cases have been described in the literature (39 reported previously and 4 additional cases presented in this article) (1, 3–14) (Table 1). Similar to the Thompson et al. series, we have found that the majority of patients with tadpole pupil are women (34/43, 79%). Likewise, they are relatively young with a median age at onset of 35.5 years old; the range is between 22 and 48 years old. More recently, however, pediatric cases (two patients age 2 years and one patient age 12 years) have been reported (7, 11, 13).

**Table 1:** Summary of tadpole pupil cases reported in the literature.

**Author**	**Sex/Age**	**Side**	**Duration of episodes**	**Frequency of episodes**	**Known trigger**	**Associated symptoms**	**Associated condition**	**Timing of HS**
Thompson	F/48	L (1xbilat)	20 s	2 clusters of 3–5 days with 8–10 attacks/days		Blurred vision /abnormal periocular sensation	No	
Thompson	F/32	R	5–10 s	From 2 to a lot of episodes/day		Blurred vision	No	
Thompson	F/47	L or R	10–15 s	From 1 to several episodes/day		Blurred vision /headache prior to episodes	Areas of intermittent sweating on the left flank and right neck	
Thompson	F/32	R	<1 min	2–3 episodes/year		Blurred vision		
Thompson	M/30	R	1 min	Several clusters of 3 h with several episodes		Light-headedness	Congenital right HS	Before tadpole dx
Thompson	F/young	L	15 min	Once Every 6 months		Blurred vision /left orbital pain	Possible migraine	
Thompson	F/37	R					Right HS	At time of tadpole dx
Thompson	F/35	R (1xL)	3–5 min	Clusters of 90 min with several episodes		Blurred vision /funny sensation in the right eye	No	
Thompson	F/27	R	A few seconds	Clusters of 2 weeks with several episodes/days		No	Right postganglionic HS	At time of tadpole dx
Thompson	F/48	R	20 s to 2 min	Clusters of 3–4 days with 15–30 episodes/day		Blurred vision	Right postganglionic HS / intermittent sweating over the medial part of the right brow	At time of tadpole dx
Thompson	F/36	R	1 min	Clusters of 3 h with 10–12 episodes		Blurred vision	No	
Thompson	F/39	R	2 h			Blurred vision	Migraine	
Thompson	F/43	L	A few seconds	Either clusters or single episodes separated with long intervals		Funny sensation in the left eye	No	
Thompson	F/33	L				Blurred vision	Probable left preganglionic HS /left tonic pupil	At time of tadpole dx
Thompson	F/30	L or R	A few min	1–3 episodes/month		Blurred vision /funny sensation in the eye	No	
Thompson	F/38	R		Very frequent episodes during 2 months		Funny sensation in the right eye	Tonic pupil in both eyes	
Thompson	F/24	L	1–2 min	Clusters of 1 to several days with episodes every 5–10 min		Light-headedness	Left postganglionic HS/probable migraine/left tonic pupil	At time of tadpole dx
Thompson	M/37	L	1–2 min	Clusters of 2–3 days with several episodes		Blurred vision /abnormal sensation in the ipsilateral face	Left postganglionic HS /migraine	At time of tadpole dx
Thompson	F/33	L or R	1 min			Blurred vision	Migraine	
Thompson	F/32	R	A few minutes	Frequent episodes		Blurred vision /funny sensation in the right eye	Right postganglionic HS /migraine	At time of tadpole dx
Thompson	F/44	L or R		Frequent episodes		Funny sensation in the eye	Left postganglionic HS/migraine	At time of tadpole dx
Thompson	M/34	R	<1 min	Clusters of several hours with up to 50 episodes		Blurred vision	Possible migraine	
Thompson	M/29	L or R or bilat	sec–min	Daily episodes since 3 years		Blurred vision /funny sensation in the eye	No	
Thompson	F/46	R	1 h				Tonic pupil bilateral	
Thompson	M/36	L	3–5 min	Clusters of 2 days with several episodes/days		Blurred vision	Probable left HS /migraine	At time of tadpole dx
Thompson	F/27	L or R	<3 min	Clusters of 2 days with 1–5 episodes/day		Funny sensation in the eye	Right HS /migraine	At time of tadpole dx
Tang	M/29	R	<1 min	Since 2–3 months, only with exercise	Strenuous exercise	Blurred vision	Right postganglionic HS / hypoplastic right internal carotid artery	Before tadpole dx
Balaggan	F/32	L	3–15 min	2 clusters of 3 days with several attacks per day in 6 months		Blurred vision / abnormal periocular sensation	Left preganglionic HS	2 months after tadpole dx
Koay	F/33	R	A few minutes	From several episodes/days to 1 episode every several weeks		Periocular sensation	Right postganglionic HS	At time of tadpole dx
Lüke	F/42	L	10 min			Blurred vision	Probable left HS	At time of tadpole dx
Lüke	F/22	L	A few minutes			Funny sensation in the left eye	Morbus Charcot-Marie-tooth / papillary druse	
Weir	M/2	L	45 min		During strabismus surgery	No	No	
Kawasaki	F/young	L	A few minutes			No	No	
Höh	F/44	R	10–15 s	Clusters of 4–15 episodes/day for several days with a break of 1 year between the 1st and 2ndcluster		Funny sensation in the right eye	No	
Vijayara-ghavan	M/19	Bilateral	Long	1 episode during recurrent seizures and hyponatremia	Recurrent seizures	No	Congenital erythropoietic protoporphyria/seizure /hyponatremia	
Hansen	F/12	Bilat (or unilat)	20 min	Initially spontaneously, then only with exercise	Physical exercise	No	Juvenile idiopathic arthritis	
Ladaique	F/39	L					Left HS/asymmetric facial flushing	2 months after tadpole dx
Deschasse	F/29	L	A few min	From several times/days to one episode/week		No	No	
Aggarwal	F/2	R	40 min	Recurrent episodes since 2 months	Following waking up	No	Congenital right HS	Before tadpole dx
Udry	F/47	R	<1 min	Clusters of 2–3 days with several episodes/day		No	Right HS	Before tadpole dx
Udry	F/41	L	<1min	Clusters of 1 week with 3–4 episodes		No	Left HS/right myopic maculopathy	Before tadpole dx
Udry	F/42	R	<1 min	Weekly episodes for 4 months, then more occasional		Blurred vision	Left tonic pupil/dizziness upon standing for 1 year with 2 syncopes	
Udry	M/32	L	1 min	3–4 episodes in 2 months		Blurred vision	Left miosis and ptosis but no demonstrated HS/retinal break	

*F, female; M, male; R, right; L, left; HS, Horner syndrome*.

Tadpole pupil is usually unilateral (40/43, 93%), and rarely bilateral (10, 13). Most of the time, the same eye is affected with each episode, though Thompson et al. found that the side of the tadpole pupil switched in 6 of 26 patients (23%). The tail of the tadpole shape (the peaked or pulled segment) can involve any segment of the iris and the affected segment can vary with each episode, leading to a changing tadpole shape in 16 of 43 patients (37%). Nine patients (20%), however, demonstrated the same distortion of shape with every episode. In 18 patients (41%), the history and observation could not definitively determine if the tadpole shape changed or not.

The duration of each episode is short, 5 min or less in 34 of 43 (79%) and 15 min or less in 37 of 43 (86%). Longer episodes are distinctly unusual. The pupillary distortion appeared spontaneously in 39 of 43 patients (91%) and was accompanied by a blurring of vision or an unusual sensation in the affected eye or on the ipsilateral side of the face in 31 of 43 (72%). The four cases of non-spontaneous pupillary distortion were temporally related to strabismus surgery, morning awakening, physical exercise, and hyponatremia with seizures: Weir et al. presented a 2-year-old boy with a single episode of tadpole pupil occurring during a non-complicated strabismus surgery and lasting 45 min (7); Aggarwal et al. described a 2-year-old girl with recurrent episodes of right tadpole pupil appearing just after waking up and lasting 40 min (11); Hansen et al. reported a 12-year-old girl with episodes of bilateral tadpole pupil provoked by physical exercise and lasting 20 min (13); and Vijayaraghavan et al. presented a 19-year-old boy with a single long episode of bilateral tadpole pupils in a context of recurrent seizures and hyponatremia (10). Of note, these four patients plus two others described by Thompson et al. had episodes of pupillary distortion longer than 20 min and lasting up to 2 h (1, 7, 10, 11, 13). Such atypical features like pediatric age, identifiable precipitating factor, and long duration suggest that they may represent a different pathophysiologic entity.

The frequency of episodes varies from one episode every few months to 10–50 episodes per day. Episodes of tadpole pupil seem to occur in clusters lasting days to weeks with a variable and often long interval between clusters. In some patients, a cluster recurs after months to years of remission and in others it subsides spontaneously without leaving any sequelae after several months to years of recurrence. Very rarely, the patient undergoes only one cluster (3/44, 7%) or experiences a single episode of tadpole pupil (3/44, 7%) without recurrence.

An ipsilateral oculosympathetic deficit, or Horner syndrome, is the most frequently associated condition and found in 20 of 43 patients with tadpole pupil (46%; 15 demonstrated and 5 probable), consistent with the finding by Thompson et al. in their series (11/26 patients, 42%) (1). For 5 out of 20 patients (25%), the Horner syndrome was present before the appearance of tadpole pupil and for 3 patients (15%), the Horner syndrome developed after the tadpole pupil. Generally, the Horner syndrome was diagnosed concomitantly with the tadpole pupil, probably because the patient never noticed a pre-existing anisocoria. Thus, it is uncertain as to whether the Horner syndrome typically precedes the tadpole pupil or vice versa. Thompson et al. described also a potential association with Adie's pupil (4/26 patients, 15%) and migraines (8/26 patients, 31%) (1). However, those associations have not been reported in subsequent studies, except for our Case 3, who presented an Adie's pupil in the contralateral eye. None of our patients had migraine.

The pathophysiologic basis of the tadpole pupil remains undefined. Several mechanisms have been proposed but none can explain all the features of the tadpole pupil.

Given the frequent association with Horner syndrome, sympathetic denervation hypersensitivity has been considered to be one mechanism of tadpole pupil. Tang et al. reported a patient who seems to support such a hypothesis. The patient had a right Horner syndrome presumably related to a hypoplastic internal carotid artery. Topical hydroxyamphetamine failed to dilate the Horner pupil in the superotemporal quadrant, indicating a segmental postganglionic sympathetic deficit (partial Horner syndrome). Following physical exercise, this segment became excessively contracted and pulled the pupil into a distorted shape. Thus, the focal area of denervation corresponded to the focal segment of iris dilator spasm, presumably due to denervation hypersensitivity to circulating catecholamines during exercise (3). Hansen et al. described a patient in whom bilateral tadpole pupil appeared following physical exercise and they suggested a focal hypersensitivity to circulating catecholamines. However, pharmacologic evidence of sympathetic denervation was not provided (13). A 2-year-old girl with a congenital right Horner syndrome who developed right tadpole pupil upon awakening was reported by Aggarwal et al. The authors suggested that morning cortisol that peaks 20 to 45 min after awakening is a possible explanation in their patient (11). The hypothesis of segmental denervation with hypersensitivity may explain tadpole pupil following exercise or waking up, and in these rare cases, the distortion of pupillary shape would be expected to be stereotypic because only the denervated segment demonstrates excessive muscular contraction. Also in these patients, one might expect topical apraclonidine to reproduce the tadpole shape as evidence of a focal hypersensitivity reaction.

Though an attractive hypothesis for these specific aforementioned cases, focal denervation hypersensitivity to circulating adrenergic substances would not explain the tadpole pupil phenomenon in those patients without an underlying sympathetic defect. In the physiologic state, we must consider that a focal contraction of the iris dilator muscle occurs either from focal neurogenic stimulation or direct non-neurogenic activation.

Thompson et al. demonstrated that the effector muscle causing the tadpole pupil is the iris dilator and not the iris sphincter. They first confirmed normal pupil light reflex during tadpole distortion as indication of normal iris sphincter function. Thereafter, they applied 10% viscous phenylephrine, a direct-acting adrenergic agonist, to a focal area of the corneoscleral limbus and reproduced the tadpole-shaped distortion (1).

The neuronal stimulus to the iris dilator is the sympathetic system. Can sympathetic neurons destined to the eye fire spontaneously and intermittently? Apparently they can. The Pourfour du Petit syndrome refers to spontaneous discharge of oculosympathetic neurons causing episodic unilateral mydriasis, often in association with lid retraction and conjunctival blanching (15). It is the clinical opposite of a Horner syndrome. Except for occasional cases of patients with headache (16–18), the Pourfour du Petit syndrome largely occurs in the setting of cranio-cervical pathology (19, 20). In the event of spontaneous oculosympathetic neuronal discharge, one possible mechanism of tadpole pupil is firing of only a few sympathetic fibers. There are, however, no experimental data to indicate that spontaneous sympathetic discharge to the eye can be focal or segmental.

Another possible mechanism for muscle spasm is non-neurogenic activation of the iris dilator muscle. The iris dilator is a smooth muscle. Unlike gastrointestinal or urinary tract smooth muscle in which contractility has autonomic pacemaker properties and gap junction excitation (21), the iris dilator is classified as a multiunit smooth muscle in which each cell is activated directly and independently, like striated muscles (22, 23). This organization allows the contraction of a focal portion of the iris dilator. Like striated muscles, the iris dilator receives neural stimulation at the neuromuscular junction (23, 24). However, unlike striated muscles, smooth muscles may respond directly to hormonal stimuli. For example, estrogen and progesterone receptors located on smooth muscles surfaces can influence muscle contractility (25–28). Sex hormone receptors have been demonstrated in the iris of rabbits (29) and humans (30). Thus, we wonder if circulating hormones, while not the principal stimulus, have the capacity to activate the iris dilator muscle. The iris is well vascularized and radially oriented vessels lie alongside the iris dilator muscle fibers. Intermittent dilator muscle stimulation by hormones may explain, in part, the propensity for tadpole pupil to occur in non-menopausal women and to vary in shape and peak from episode to episode. Thompson et al. had noted that 4 of 21 women (19%) reported tadpole episodes during or around menstrual periods (1). We recognize that the hormonal hypothesis fails to explain the appearance of tadpole pupil in men, children, and post-menopausal women as well as the brief timing of tadpole pupil. It is interesting to note that when tadpole pupil occurs in men, there is a greater association with an external trigger or underlying pathology (3/9, 33%). These have included hypoplastic internal carotid artery (3), strabismus surgery (7), and hyponatremia (10). Further investigations are needed to clarify any potential role of hormones in the development of a tadpole pupil.

The iris dilator, being a multiunit smooth muscle, does not possess autonomic pacemaker contractility. However, Lee et al. have shown that *in vitro* muscle fibers of the vas deferens, also a multiunit smooth muscle, develop spontaneous contractions after denervation (31). Thus, spontaneous contractility of a denervated iris dilator muscle can be a predisposing factor for why patients with Horner syndrome have a greater risk for developing tadpole pupil compared to the general population. It remains unclear why tadpole pupil, as a manifestation of denervation, is so rare among all cases of Horner syndrome.

How might the proposed causes of tadpole pupil have clinical implications for treatment?

If hormonal influences are clearly associated with the development of clusters of tadpole pupil, for example, occurrence during menses, it may be possible that systemic regulation of estrogen, e.g., oral contraceptive agents or estrogen patch, offers a potential solution to modulating or halting the clusters. In any event, any type of hormonal manipulation must be discussed as empiric therapy with the patient's general internist or gynecologist.

As focal iris dilator spasm is the common final effector to produce a tadpole pupil, application of a topical alpha-1 adrenergic antagonist, like brimonidine, during the attack or during a cluster period can be expected to reverse the tadpole pupil and stop further episodes. Alpha-1 adrenergic antagonists induce a pharmacologic Horner syndrome. In anecdotal trials, we have found that treating a patient with topical brimonidine did abort further episodes of tadpole pupil, as well as another episodic pupillary phenomenon called a benign episodic mydriasis (RK, personal experience and communication). It is not clear if brimonidine will have a similarly favorable effect on tadpole pupil in patients who already have an oculosympathetic defect (Horner syndrome).

Finally, we wish to point out that most persons with tadpole pupil are not impaired by the occasional episodes and thus treatment is generally not necessary.

In conclusion, the tadpole pupil may be a phenomenon having multiple pathophysiologic mechanisms and associations. Because of the high frequency of association with Horner syndrome, we suggest that all persons with tadpole pupil undergo pharmacologic testing for an oculosympathetic deficit. Without or with Horner syndrome, the tadpole pupil appears to be a benign condition, which itself does not lead to any chronic sequelae nor portends more serious underlying pathology.

## Author Contributions

MU, RK, FS, and AK contributed to conception of the manuscript. AK and MU performed literature review and drafted the manuscript. MU created the table and formatted the figures. AK, RK, and FS provided the original data. AK, MU, RK, and FS revised the draft and reviewed the figures. All authors reviewed and approved the final manuscript.

### Conflict of Interest Statement

The authors declare that the research was conducted in the absence of any commercial or financial relationships that could be construed as a potential conflict of interest.
